# Academic Tangping scale for college students in China: scale development, validation and application

**DOI:** 10.1038/s41598-026-38759-2

**Published:** 2026-02-09

**Authors:** Shan Lu, Yanchao Yang, Wangze Li, Bosheng Jing, Yuanfang Guo

**Affiliations:** 1 Qinggong College, North China University of Science and Technology, Tangshan, Hebei Province 063200 People’s Republic of China; 2 Institute of International Language Services Studies, Macau Millennium College, Macau SAR, 999078 People’s Republic of China; 3 College of Materials Science and Engineering, North China University of Science and Technology, Tangshan, Hebei Province 063200 People’s Republic of China; 4 School of Humanities and Languages, The University of New South Wales, Sydney, 2055 Australia; 5 Faculty of Child Development and Education, Zhejiang Normal University, Jinhua, Zhejiang Province 321004 People’s Republic of China

**Keywords:** Tangping, Lying flat, Scale development, Scale validation, Scale application, Human behaviour, Health care, Public health

## Abstract

In light of the universality of the Tangping phenomenon and the lack of measurement tools, this study aims to develop Academic Tangping Scale for assessing this phenomenon among for college students in China. A total of 644 valid Chinese university students participated in the research. Exploratory factor analysis supported the extraction of three dimensions, namely affective, cognitive and behavioral dimensions, and confirmatory factor analysis indicated a good model fit, further confirming the scale’s dimensional structure. High Cronbach’s alpha coefficients across the subscales further affirm its robustness as a psychometrically sound measurement instrument. Evidence for convergent and discriminant validity was rigorously substantiated, and significant Pearson correlations with the existing Lying Flat Tendency scale demonstrated criterion-related validity. Finally, the scale’s practical applicability was supported by significant correlations with depression, anxiety, and stress, indicating meaningful associations with students’ mental health outcomes. The scale acknowledges limitations and proposes corresponding suggestions. Overall, the scale demonstrates strong psychometric properties, providing a reliable tool to assess the Academic Tangping and support targeted interventions and research on student mental health.

**Supplementary Information:**

The online version contains supplementary material available at 10.1038/s41598-026-38759-2.

## Introduction

In recent years, the concept of “Tangping”, or “lying flat, or quiet quitting” has gained significant attention, becoming a trending topic on social media and igniting widespread discussions, particularly among young people in mainland China^1–5^ and even in Hong Kong SAR^6,7^. “Tangping” refers to a conscious decision to reject the relentless pursuit of material success, opting instead for a minimalist lifestyle focused on doing the bare minimum for survival^8,9^. This trend reflects a growing disillusionment among youth, who increasingly push back against the pressures of intense work culture and societal demands for status and wealth^10,11^.

While Tangping reflects a broader academic attitude aimed at reducing societal pressure and personal stress, it differs from academic-specific phenomena such as academic burnout and academic procrastination, which are more directly tied to students’ experiences within educational settings. Academic burnout syndrome, also referred to as “educational burnout” or “burnout in education,” is a form of burnout that specifically affects individuals in the educational domain, including teachers, researchers, and students^12^. It is characterized by a state in which students, subjected to prolonged academic stress and heavy workloads, experience exhaustion, gradually lose interest in school tasks and activities, and exhibit decreased motivation for learning^13^. Academic procrastination, on the other hand, is a recurring behavioral pattern in students’ academic life, involving the deliberate postponement or delay of required tasks despite impending deadlines^14^.

The Chinese National Language Resources Monitoring and Research Center, an institution affiliated with the Education Ministry of China, listed the word as one of the 10 most popular memes for 2021 in the Chinese Internet^15^. The term “Tangping” still continues to maintain its status as a trending topic online, with frequent discussions surrounding it. On Douyin, the average daily search volume has reached 172,000^16^, illustrating ongoing reflections and resistance among young people regarding lifestyle choices and societal pressures. Many users share their personal “Tangping” experiences, recounting their thoughts and feelings about this lifestyle choice. Such discussions not only illuminate the genuine sentiments of young people navigating a high-pressure society but also create a platform for expressing their desires for balance and fulfillment in the face of societal expectations. This cultural movement represents a conscious rejection of the relentless pursuit of success, particularly within the competitive environments of academic and professional life, under which young individuals are increasingly embracing a lifestyle characterized by minimalism and a deliberate withdrawal from these pressures.

Similar trends have emerged globally. In Japan, the “freeter” phenomenon^17^ describes individuals who opt for part-time or temporary work over traditional careers. South Korea’s “N-po generation”^18,19^ reflects a cohort disillusioned with societal norms related to employment and marriage. Meanwhile, in the UK, the term “NEET” (Not in Education, Employment, or Training)^20^ highlights a segment of youth disengaged from the conventional paths of education and work. These trends illustrate a broader global movement among young people, who are increasingly questioning established norms and seeking alternative lifestyles that prioritize personal well-being over societal pressures. This growing sentiment reflects a collective desire for a more balanced and fulfilling life amidst the challenges of modern society. Overall, this phenomenon appears to be widespread, indicating that contemporary young people are increasingly breaking away from traditional societal expectations in their academic, career, and lifestyle choices, and are seeking greater autonomy and inner peace.

The rise of “Tangping” can be attributed to various factors. In the educational realm, issues such as flaws in current education and talent evaluation systems and the over-utilitarian focus of education have played a significant role^21^. For instance, the intense competition in China’s education system, often leading to high-stakes exams, creates immense pressure on students, pushing some to reject traditional success metrics. On the societal front, factors include a lack of social mobility^21,22^, long working hours, elevated stress levels, and limited personal time^7^, along with increasing income inequality, soaring housing costs^22^. For instance, the prevalent “996” work culture (9 AM to 9 PM, six days a week) in many Chinese companies fosters burnout^23^, work-life imbalance^24^, and psychological distress^25^, leading individuals to reject overtime and adopt a lifestyle focused on minimal effort, prioritizing their well-being over traditional career ambitions.

The rise of Tangping poses significant risks at multiple levels, as it negatively impacts individual mental health, undermines organizational performance, and hinders national innovation and economic growth. First, Tangping behaviors have notable implications for mental health for individuals. For instance, Tangping behaviors are positively correlated with depressive symptoms^26^, suggesting that this disengagement from societal expectations can lead to increased feelings of depression among individuals. In addition, the tendency to adopt a Tangping attitude is negatively correlated with overall mental health^27^, indicating that this lifestyle choice may contribute to a decline in mental well-being, as it often involves a lack of engagement and purpose. In addition to its implications for individual mental health, the phenomenon of Tangping also exerts negative effects on organizational dynamics. This behavior can contribute to a diminished sense of ownership among employees^28^, as individuals may withdraw from their roles and responsibilities. Such disengagement can subsequently undermine organizational performance^29^. Furthermore, Tangping can also have significant implications for national innovation^10^. This phenomenon may lead to a decline in workforce motivation and creativity, as individuals prioritize personal well-being over ambition and productivity. When a substantial portion of the population adopts a passive mindset, it can hinder collaborative efforts and reduce the drive for innovative solutions. Consequently, this lack of initiative may stifle economic growth and technological advancement, ultimately affecting the country’s competitiveness in a global landscape that increasingly values innovation. Therefore, researching Tangping is critically important, as it has the potential to uncover effective strategies for mitigating its negative impacts on individuals, organizations, and society as a whole. This research can help improve individual mental health by identifying coping mechanisms and support systems to address depressive symptoms. Additionally, it can enhance organizational performance by informing practices that foster employee engagement and a sense of belonging. On a national level, such research can guide policies aimed at boosting innovation and economic growth, ultimately contributing to a more resilient and competitive society.

To effectively address these challenges, developing a measurement scale specifically focused on “Tangping” is crucial. Such a scale would provide a quantitative research tool to capture the prevalence and dimensions of this phenomenon, thereby facilitating a deeper understanding of its antecedents and consequences across individual, organizational, and national levels. Furthermore, the phenomenon of Tangping has parallels in other regions, such as the freeter, N-po generation, and NEET. Therefore, this scale would also hold significance for other countries, offering insights into similar trends and behaviors.

However, current research faces limitations in measuring “Tangping”. First, one utilize a single-item self-report measure^30^, such as “To what extent are you feeling positive toward lying flatism?” This simplistic design fails to capture the complexity of the phenomenon, as it does not account for various dimensions or nuances of feelings related to Tangping. The reliance on a single question limits the depth of understanding regarding individual attitudes and experiences. Secondly, another research employs scenario questions to measure attitudes toward Tangping^31^. While this method can provide context, it may suffer from limitations such as response bias, where individuals might not react authentically to hypothetical situations, leading to results that do not accurately reflect real-world attitudes and behaviors. Additionally, the absence of reliability and validity gave rise to concerns about the robustness of their findings. Thirdly, other study considers Tangping solely as a behavioral dimension^32^. Furthermore, the existing multidimensional quiet quitting scale^33^ was developed and validated based on emotional and behavioral dimensions to assess employee disengagement in the workplace. However, this study argues that Tangping should not be considered solely as a behavior; instead, it should encompass three dimensions: behavioural, affective, and cognitive. Recognizing these three dimensions is essential because behavior reflects an individual’s active choice to withdraw from societal pressures and expectations. The cognitive dimension involves an individual’s assessment of societal values and personal priorities, influencing their decision to adopt this lifestyle. Additionally, the affective dimension captures the feelings of disillusionment, relief, or empowerment that individuals experience when choosing to Tangping. Therefore, when developing a scale to measure Tangping, it is crucial to include all three dimensions to ensure a comprehensive understanding of this complex phenomenon.

What’s more, while Patel et al.’s scale and another scale^34–38^ are specifically designed for work contexts, the present study aims to develop and validate a scale specifically for academic contexts. Differences in target population and environment may lead to distinct manifestations, indicating that workplace-oriented scales may not fully capture students’ academic Tangping.

To conclude, limitations in previous measurement tools regarding Tangping—including the reliance on single-item measures, potential response biases, lack of reliability and validity evidence, inappropriate dimensional coverage, and differences in contextual settings—underscore the necessity of developing a reliable and valid scale specifically tailored to the academic context. Therefore, the goal of this study is to develop a scale for measuring Tangping among university students. Specifically, this research aims to create a scale that captures the aspects of Tangping, validate its reliability and validity using Classical Test Theory methods, and explore the relationship between Tangping and depressive symptoms to show how the scale can be used to understand its relation with mental health issues indicated by anxiety, stress and depression.

##  Methods

### Participants

Participants’ responses were collected using an online questionnaire administered via Wenjuanxing, a widely used online survey platform in China. The survey link was distributed to college students, and participation was voluntary. Convenience sampling was employed for participant recruitment. The participants in this study came from several universities in northern China, with a total of 650 questionnaires collected. Among these 650 individuals, three participants did not provide their age, and three others had ages that were clearly inconsistent with student status, including one participant aged 45 and another two under 16. Therefore, the final number of valid participants was 644. The mean age of the participants was 19.304 years (standard deviation = 1.575). Most participants were from NCUST, totaling 633 individuals, which accounts for 98.29%. The majority of the students were undergraduates, with 610 individuals, representing 94.72%. Students from rural backgrounds numbered 477, making up 74.07%; and 478 participants were male, constituting 74.22%. The data were randomly divided into two groups: 326 individuals for Exploratory Factor Analysis (EFA) and 318 for Confirmatory Factor Analysis (CFA), as detailed in Table 1.

**Table 1:** Demographic information.

Demographic variable	Group	Total		EFA dataset		CFA dataset	
		N	%	N	%	N	%
Gender	Male	478	74.22%	238	73.01%	240	75.47%
	Female	166	25.78%	88	26.99%	78	24.53%
Birthplace	Rural	477	74.07%	236	72.39%	241	75.79%
	Urban	167	25.93%	90	27.61%	77	24.21%
Institution	NCUST	633	98.29%	320	98.16%	313	98.43%
	Others	11	1.71%	6	1.84%	5	1.57%
Educational background	Undergraduate	610	94.72%	314	96.32%	296	93.08%
	Vocational Student	2	0.31%	1	0.31%	1	0.31%
	Graduate	32	4.97%	11	3.37%	21	6.60%

Due to submission anonymity requirements, the full name of “NCUST” is not displayed in the text.

### Instruments

#### Academic Tangping scale for college students in China

##### Conceptualizing and operationalizing academic Tangping

Since there is currently no standardized definition of “Academic Tangping” and most research on Tangping has primarily focused on the work context^31,39–42^, it is essential to establish a clear definition of Academic Tangping to understand its implications in educational settings.

In the stages of conceptualizing and operationalizing Academic Tangping, the decision to structure the construct around three dimensions was grounded in existing theoretical guidance. A growing body of literature has consistently conceptualized Tangping as an attitude rather than merely a behavioral pattern or a sociocultural stance^22,31,43–46^. These studies converge in viewing tangping as an attitudinal stance that individuals develop in response to academic or societal pressures, therefore, the present study builds upon this shared understanding in defining Academic Tangping. Guided by literature, the current study adopts the classical ABC model of attitudes as the theoretical foundation for conceptualization^47^. The ABC model, which delineates the affective, cognitive, and behavioral components of attitudes, offers a well-established framework for translating the attitudinal nature of Academic Tangping into measurable dimensions. Accordingly, this model informed both the conceptual definition of Academic Tangping and the generation of the initial item pool.

In this study, Academic Tangping can be defined as a mindset and lifestyle choice among students characterized by a conscious decision to disengage from traditional academic pressures and societal expectations. This disengagement reflects a broader affective response to stress and burnout, a cognitive re-evaluation of the value placed on academic success, and specific behavioral changes that prioritize well-being over competitive achievement.

##### Generation of item pool

Item generation for the scale is based on semi-structured interviews conducted with four university students, recommended by their Ideological and Political Education Counselor for exemplifying these characteristics. Based on this ABC model of attitude, the research team developed an interview protocol. The interview questions primarily focus on exploring the specific behaviors, affections, and cognitions that represent Tangping. Through these questions, the study aims to gain deeper insights into students’ perspectives on Tangping to generate relevant scale items. The interview lasted 50 about minutes. After transcribing the interview recordings, the research team initially performed open coding to identify key phrases and concepts related to Tangping. These initial codes included affective responses, specific behaviors, and psychological cognitions expressed by the students. For instance, the team identified affective states such as calmness and avoidance of anxiety, behavioral patterns like the refusal to invest additional time and effort, and personal views on the Tangping phenomenon, including perceptions of limited abilities and ineffective effort. The research team then organized these initial codes into broader themes: affective, behavioral, and cognitive.

##### Expert feedback

To establish expert validity, eight experts were consulted, all of whom were university ideological and political counselors. Their selection was based on their expertise in student behavior, as well as their familiarity with the Tangping phenomenon. They assessed the representativeness, relevance, and clarity of the dimensions and items, providing scores on a scale of 1 to 4 for each aspect. Finally, the research team calculated the Item-Level Content Validity Index (I-CVI) and the Scale-Level Content Validity Index (S-CVI). The I-CVI was calculated by the number of experts providing a score of 3 or 4 divided by the total number of experts^48^, with values greater than 0.79 considered acceptable. The sum the I-CVI scores of all items divided by the total number of items (average of the I-CVI scores for all items across all experts) = S-CVI/Ave. The S-CVI value of 0.8 or higher is considered acceptable^49^. The results showed that, except for the representativeness I-CVI of 0.88 for E2, E4, and C1, all other items received an I-CVI of 1 for representativeness. All items also achieved an I-CVI of 1 for clarity and relevance. Additionally, the S-CVI for representativeness was 0.98, while the S-CVI for clarity and relevance was 1. Consequently, the items on the scale were neither reduced nor modified.

In conclusion, the Academic Tangping Scale for College Students in China is designed to measure three proposed key dimensions of Tangping behavior: affective, behavioral, and cognitive. Each dimension consists of five items that reflect specific aspects of the Tangping phenomenon. The affective dimension assesses students’ feelings related to Tangping (e.g., *I am very satisfied with my current study situation; I feel calm and do not want to add excessive pressure and anxiety to myself*). The behavioral dimension focuses on observable behaviors associated with Tangping (e.g., *I meet the academic requirements but will not invest extra time to improve my grades*). The cognitive dimension assesses students’ perceptions and beliefs about their academic abilities and the effort they invest (e.g., I believe that no matter how hard I try, I cannot achieve my ideal goals, so I choose not to push myself anymore). The scale employs a 5-point Likert scoring system, where 1 represents “Strongly Disagree” and 5 represents “Strongly Agree,” allowing for nuanced responses to each item. The development process was presented in the Results section. The complete set of the scale is presented in Appendix 1.

#### “Lying Flat” tendency scale for the youth

The “Lying Flat” Tendency Scale for Youth^32^ is a measurement tool developed to systematically evaluate an individual’s tendency to adopt a “lying flat” lifestyle. Comprising one dimension with six items, the scale provides a focused evaluation of this phenomenon. It has undergone rigorous cross-validation for reliability and validity across diverse samples in China. The internal consistency coefficient in the current study, measured by Cronbach’s α, is 0.957, indicating strong reliability. A higher total score reflects a greater tendency to embrace this lifestyle.

#### Depression anxiety stress scales

The Depression Anxiety Stress Scales-21-N^50^ was adapted based on the 21-item Depression Anxiety Stress Scales^51^. It includes Anxiety (7 items), Depression (6 items) and Stress (8 items). The choice of this scale is based on three main reasons. Firstly, research has demonstrated a positive correlation between the Tangping attitude and depressive symptoms^26^, while adopting a Tangping mindset is negatively associated with overall mental health^27^. Secondly, the scale facilitates the simultaneous measurement of anxiety, depression, and stress, which increases the thoroughness of the assessment. Lastly, the scale has proven high reliability (Cronbach’s alphas 0.79 for Anxiety, 0.91 for Stress, and 0.93 for Depression) and validity, and it has been cited in numerous research articles^52,53^, highlighting its importance and dependability in related studies.

### Ethical considerations

Prior to the research, an Institutional Review Board (IRB) application was submitted to Medical Ethics Committee of North China University of Science and Technology and approved with IRB No. SQ-2,024,008. All procedures involving human participants were performed in accordance with the ethical standards of the Declaration of Helsinki and the relevant national regulations. This ensures that the study adheres to ethical standards and protects participants’ rights. Informed consent was obtained from all participants. Specifically, the survey includes an embedded informed consent form that outlines the purpose of the study, the researcher’s contact information, and other relevant details. Participants shall click the “Agree to Participate” option to proceed, ensuring they are fully informed before participating. To safeguard participants’ privacy, no identifiable information was collected during the study, which protects individual identities and conforms to ethical guidelines regarding confidentiality. All participants have the right to withdraw from the study at any time without facing any penalties, which guarantees that participation is entirely voluntary and respects the autonomy of individuals involved. All collected data is stored on password-protected computers, accessible only to the researchers.

### Analytical procedure

This study utilizes a multi-step design to ensure rigorous and reliable findings. It started with conceptualization and operationalization of Academic Tangping, followed by item generation, and expert feedback.

Next, item analysis was conducted using JASP^54^, including item discrimination, item-total correlations, and Cronbach’s alpha if an item was deleted, to evaluate the impact of each item on the scale’s internal consistency. Following this, exploratory factor analysis (EFA) was conducted with JASP^54^ using the varimax rotation method to elucidate the scale’s structure and dimensions. A threshold of 0.40 for factor loadings was set to ensure that items significantly contributed to their respective factors. Communalities were assessed, with values above 0.40 considered desirable. Items with significant cross-loadings were carefully evaluated, as they could complicate interpretation and required reconsideration or removal.

Then, CFA was conducted using the CB-SEM module of SmartPLS^55^ to assess various forms of validity, including structural validity, convergent validity, and discriminant validity, using indicators such as model fit indices, namely, Comparative Fit Index (CFI), Tucker-Lewis Index (TLI) Root Mean Square Error of Approximation (RMSEA), Standardized Root Mean Square Residual (SRMR), with thresholds of CFI and TLI greater than 0.90, RMSEA less than 0.08, and SRMR less than 0.05 for structural validity^56,57^; for convergent validity, average variance extracted (AVE) greater than 0.50 and composite reliability (CR) greater than 0.70^58^; and the Fornell-Larcker criterion for discriminant validity^59^, where the correlations between constructs should be less than the square root of their respective AVEs.

Additionally, by calculating the Pearson correlation coefficient using JASP^54^ between the Academic Tangping scale and the “lying flat tendency” scale, the current study gathered robust evidence to support the criterion-related validity of the instrument.

Finally, the scale was specifically applied to assess its effectiveness by calculating the Pearson correlation coefficient using JASP^54^ in exploring the relationship between Tangping and various psychological constructs, including depression, stress, and anxiety. This application of the Academic Tangping Scale aimed to evaluate its utility in understanding how the phenomenon of “Tangping” interacts with these mental health factors.

## Results

### Descriptive results

Descriptive statistics and Pearson product–moment correlation analyses were conducted. As shown in Appendix 2, the mean scores of all items ranged from 2.58 to 3.48, with standard errors ranging from 0.057 to 0.064. The standard deviations ranged from 1.034 to 1.159, indicating a moderate level of variability in participants’ responses across items.

In addition, inter-item correlations were examined. All correlation coefficients were statistically significant at the 0.01 level (two-tailed), with values ranging from.148 (between Item C4 and Item A3) to 0.845 (between Item C4 and Item C5).

### Item analysis

#### Item discrimination

We calculated the total score by summing the item scores, designating the top 27% as the high-score group (Total Score = 50) and the bottom 27% as the low-score group (Total Score = 40). An independent samples t-test revealed significant differences for each item at a 95% confidence interval, indicating strong discriminative power.

#### Item-total correlations

Subsequently, the author employed item-total correlations to assess the relationship between each item and the overall scale score. The results indicated that the Pearson correlation coefficients between the items and the total score ranged from 0.612 to 0.823, demonstrating a strong positive relationship between the items and the overall score.

#### Reliability statistics

The analysis revealed a Cronbach’s alpha coefficient of 0.938 for the 15 items on the scale, indicating excellent internal consistency. Furthermore, the assessments of “Corrected Item-Total Correlation” and “Cronbach’s Alpha if Item Deleted” confirmed that all items were essential, as removing any single item would not significantly enhance internal consistency.

### Exploratory factor analysis

Prior to performing the exploratory factor analysis, the Kaiser-Meyer-Olkin (KMO) test and Bartlett’s test of sphericity were conducted. The KMO value was 0.918, suggesting that the data were appropriate for factor analysis. Bartlett’s test yielded a result of 4465.420 (*df* = 105), with a significance level of less than 0.001, further confirming the suitability of the analysis.

Subsequently, the exploratory factor analysis results presented in Table 2, using the Varimax rotation method, indicated that all item factor loadings were above 0.4, with no cross-loadings exceeding this threshold. Additionally, all items demonstrated communalities greater than 0.6, suggesting that none needed to be removed. The analysis, based on the criteria of eigenvalues greater than 1 revealed three factors, aligning with the proposed three-dimensional structure.

In detail, the first factor identified was Cognitive, which accounted for 28.985% of the variance, with a Cronbach’s Alpha of 0.949. The second factor was Affective, explaining 25.120% of the variance, with a Cronbach’s Alpha of 0.897. The third factor was Behavioral, accounting for 24.153% of the variance, with a Cronbach’s Alpha of 0.933.

**Table 2:** Rotated component matrix.

Item	Cognitive	Affective	Behavioral	Cronbach’s Alpha	% of Variance	Communalities
A1	0.168	**0.773**	0.193	0.897	25.120	0.663
A2	0.230	**0.698**	0.316			0.639
A3	0.031	**0.863**	0.251			0.809
A4	0.061	**0.849**	0.223			0.774
A5	0.090	**0.815**	0.184			0.706
B1	0.358	0.338	**0.727**	0.933	24.153	0.771
B2	0.357	0.293	**0.777**			0.817
B3	0.310	0.288	**0.804**			0.825
B4	0.390	0.225	**0.776**			0.805
B5	0.227	0.377	**0.748**			0.754
C1	**0.791**	0.164	0.366	0.949	28.985	0.786
C2	**0.854**	0.135	0.259			0.814
C3	**0.907**	0.138	0.191			0.879
C4	**0.883**	0.084	0.264			0.856
C5	**0.862**	0.081	0.300			0.839

Note: Extraction Method: Principal Component Analysis, Rotation Method: Varimax with Kaiser Normalization, Rotation converged in 5 iterations.

Factor loadings > 0.4 are highlighted in bold.

### Structural validity

Next, a Confirmatory Factor Analysis (CFA) was performed using the second dataset to verify whether the existing factor structure aligns with the collected data. The results in Table 3 indicate a χ² of 272.992 with 87 degrees of freedom, resulting in a χ²/df ratio of 3.138. Additionally, the TLI is 0.947, the CFI is 0.956, the RMSEA is 0.082, and the SRMR is 0.052. Although the SRMR and RMSEA slightly exceed the recommended standards, they remain within acceptable limits. These indicators suggest that the model fits well, supporting the hypothesized factor structure.

**Table 3:** Fit results.

Index	χ2	df	χ2/df	RMSEA	SRMR	TLI	CFI
Model fit	272.992	87	3.138	0.082	0.052	0.947	0.956
Recommended Cut-off Value			1–6	< 0.080	< 0.050	> 0.900	> 0.900

### Convergent validity

**Fig. 1:**
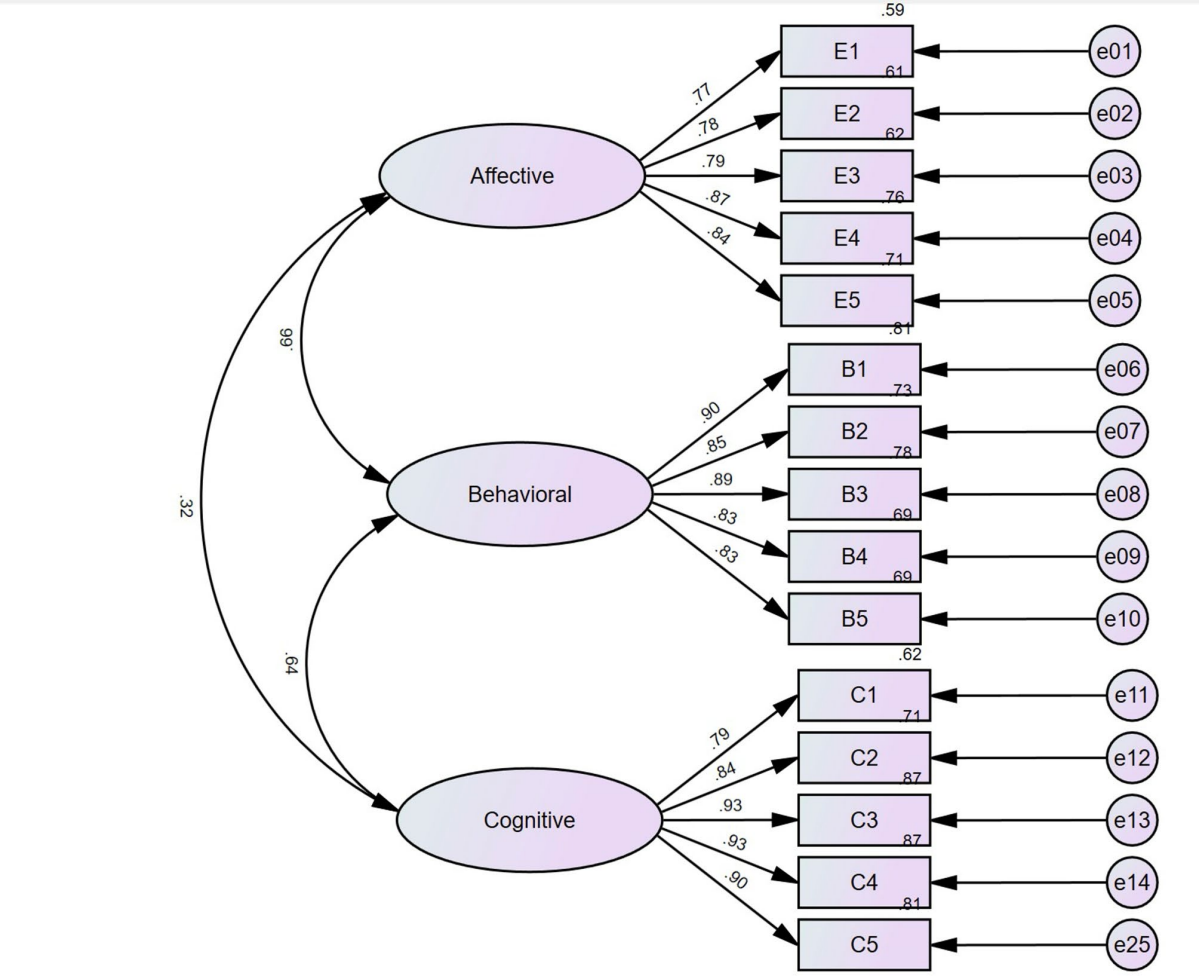
Three-factor structure of academic Tangping scale.

The analysis results for convergent validity presented in Table 4; Fig. 1 demonstrate that the standardized loadings ranged from 0.770 to 0.934, indicating robust relationships between the observed variables and their respective factors. The Composite Reliability (CR) values ranged from 0.906 to 0.945, reflecting good internal consistency among the items. Additionally, the Average Variance Extracted (AVE) values ranged from 0.660 to 0.777, all exceeding the recommended thresholds. Collectively, these findings confirm that the Academic Tangping Scale exhibits strong convergent validity.

**Table 4:** Convergent validity results.

Item	Factor	UnSTd Estimate	Std Estimate	S.E.	C.*R*.	*P*	CR	AVE
A1	Affective	1.000	0.770				0.906	0.660
A2		1.092	0.780	0.075	14.563	***		
A3		1.087	0.790	0.074	14.780	***		
A4		1.188	0.872	0.072	16.616	***		
A5		1.156	0.845	0.072	16.012	***		
B1	Behavioral	1.000	0.901				0.935	0.741
B2		0.943	0.855	0.043	21.880	***		
B3		0.950	0.886	0.040	23.696	***		
B4		0.906	0.832	0.044	20.669	***		
B5		0.904	0.828	0.044	20.483	***		
C1	Cognitive	1.000	0.789				0.945	0.777
C2		1.095	0.844	0.063	17.271	***		
C3		1.152	0.934	0.058	19.933	***		
C4		1.199	0.930	0.061	19.809	***		
C5		1.200	0.899	0.064	18.865	***		

UnSTd Estimate = Unstandardized Estimate, Std Estimate = Standardized Estimate, S.E. = Standard Error, C.R. = Critical Ratio, CR = Composite Reliability, and AVE = Average Variance Extracted.

### Discriminant validity

Discriminant validity was assessed using the Fornell-Larcker criterion, which compares the Average Variance Extracted (AVE) of each construct to the squares of their correlations with other constructs, ensuring that the AVE is greater than the squared correlation values. As illustrated in Table 5, all constructs satisfied this criterion, indicating that each construct is distinct and that the measures are capturing unique dimensions of the underlying theoretical framework. These findings support the discriminant validity of the constructs in this study.

**Table 5:** Fornell-Larcker results.

	Affective	Behavioral	Cognitive
Affective	0.812		
Behavioral	0.663***	0.861	
Cognitive	0.321***	0.644***	0.881

The square roots of the AVE values are displayed on the diagonals in bold, while the non-diagonal elements represent the correlations between the latent variables.

### Criterion-related validity

Following this, a criterion-related validity assessment was conducted to examine the relationship between the Academic Tangping Scale and the lying flat tendency questionnaire. This analysis employed the lying flat tendency as a criterion measure to evaluate the extent to which the Academic Tangping Scale correlates with this relevant construct. The results in Table 6 revealed significant correlations between all three dimensions of the Academic Tangping Scale, as well as the total score, and the lying flat tendency. The Pearson correlation coefficients ranged from 0.266 to 0.795, indicating a strong relationship and supporting the criterion-related validity of the Academic Tangping Scale.

**Table 6:** Criterion-related validity results.

Variable	1	2	3	4	5
1. Affective	—				
2. Behavioral	0.612	—			
3. Cognitive	0.314	0.634	—		
4. Tangping	0.763	0.906	0.804	—	
5. Lying flat tendency	0.266	0.558	0.795	0.667	—

Note: significant at p < 0.001.

### Application of academic Tangping scale

The scale was specifically applied to assess its effectiveness in exploring the relationship between Tangping and various psychological constructs, including depression, stress, and anxiety^50^. The results in Table 7 demonstrated that the Pearson Correlation Coefficients between Tangping, its sub-dimensions, and depression, stress, and anxiety were all significant, indicating the applicability of the Tangping scale.

**Table 7:** Pearson correlation coefficients between Tangping, its sub-dimensions, and depression, stress, and anxiety.

Factor/Construct	M	SD	1	2	3	4	5	6	7
1. Anxiety	2.179	0.723	--						
2. Depression	1.960	0.799	0.719^**^	--					
3. Stress	2.318	1.068	0.758^**^	0.849^**^	--				
4. Affective	3.211	0.841	0.122^*^	0.136^*^	0.141^*^	--			
5. Behavioral	3.002	0.868	0.226^**^	0.310^**^	0.308^**^	0.612^**^	--		
6. Cognitive	2.585	0.942	0.438^**^	0.480^**^	0.501^**^	0.314^**^	0.634^**^	--	
7. Tangping	2.932	0.728	0.325^**^	0.382^**^	0.392^**^	0.763^**^	0.906^**^	0.804^**^	--

**. Correlation is significant at the 0.01 level (2-tailed).

Correlation is significant at the 0.05 level (2-tailed).

M = mean; SD = standard deviation.

## Discussion

The Academic Tangping Scale captures the academic “lying flat” phenomenon through three dimensions: affective, behavioral, and cognitive. Results from multiple stages of validation demonstrate that the scale exhibits strong reliability and validity, affirming its effectiveness as a measurement tool.

The adoption of the classical ABC model of attitudes to structure Academic Tangping is theoretically justified. Previous research has increasingly conceptualized Tangping as an attitudinal stance^22,31,43–46^, highlighting the importance of capturing its affective, cognitive, and behavioral aspects. The ABC model of attitudes^47^, which systematically specifies these three components, provides a well-established framework for operationalizing such attitudinal constructs. To begin with, the affective dimension includes items that emphasize emotional satisfaction, highlighting how individuals prioritize contentment over stress. For instance, statements such as, “I feel very satisfied with my current learning state and do not want to add excessive pressure and anxiety to myself,” reflect a conscious choice to seek emotional well-being rather than succumbing to external academic pressures. This dimension illustrates a profound understanding of the importance of maintaining mental peace amidst academic demands. This affective dimension is consistent with previous research^33^, which likewise conceptualizes it as individuals’ emotional responses to exerting effort beyond basic or minimum requirements.

Furthermore, the behavioral dimension reflects a laid-back attitude towards academic responsibilities, as evidenced by items indicating that individuals meet essential requirements without striving for higher grades. For example, the statement, “I complete academic requirements but do not invest extra time to improve my grades,” conveys a clear intention to fulfill obligations without overexerting oneself, underscoring a preference for balance over relentless ambition. This behavioral pattern is consistent with previous research, which has similarly described a tendency among students to prioritize basic academic compliance while minimizing excessive performance-oriented effort^33^.

Lastly, the cognitive dimension encompasses beliefs about ability and effort, with items that reveal skepticism about the effectiveness of hard work. For instance, statements like, “I believe my abilities are limited, and effort cannot change my situation,” capture a mindset that views personal achievement as largely dictated by external factors rather than individual effort. This perspective highlights a resignation to one’s circumstances, reinforcing the “lying flat” philosophy.

Additionally, the study validated the scale’s application by examining its correlation with anxiety, stress, and depression. This indicates that the Academic Tangping Scale is not only a reliable measure of the “lying flat” mindset but also relevant in understanding its impact on mental health. Overall, these findings provide a solid foundation for further research and interventions related to the challenges posed by the academic “lying flat” mindset.

## Limitations and suggestions

This study acknowledges several limitations that may impact the findings. Firstly, while data were collected from students across various universities in Northern China, a significant portion of the responses originated from a single institution. This concentration may limit the representativeness of the sample, as students from one institution may share similar backgrounds, academic environments, and attitudes toward learning, which might not fully capture the diversity of the broader student population. Consequently, the findings may be influenced by institution-specific cultural or educational practices, potentially biasing estimates of Academic Tangping levels and the relationships among its dimensions. To address this limitation, future research should adopt multi-stage stratified sampling across universities in different regions of China (e.g., eastern, central, and western), covering institutions of varying types and sizes. Such a design would ensure greater heterogeneity in demographic characteristics, academic experiences, and learning environments, thereby enhancing the generalizability and external validity of the findings. Additionally, since the Tangping phenomenon has been observed in various countries—for example, the freeter in Japan, the N-po generation in South Korea, and NEETs in the U.K.—future studies should consider validating the Academic Tangping Scale across different cultural contexts. Such cross-cultural validation would allow researchers to examine whether the underlying dimensions of Academic Tangping (cognitive, emotional, and behavioral) are universally applicable or culture-specific, and whether scale items require adaptation to reflect cultural nuances.

Secondly, although various demographic variables were collected, the proportions of participants in the randomly assigned groups varied significantly. This discrepancy may influence the results and their interpretation; consequently, measurement invariance was not assessed. In future studies, it is crucial to minimize differences in group sizes to enable a more accurate assessment of measurement invariance.

Third, the cross-sectional design of this study prevents conclusions about causality or the temporal direction of the observed associations between mental health indicators (stress, anxiety, depression) and Academic Tangping. It remains unclear whether mental health problems contribute to Tangping, whether Tangping influences mental health, or whether both are affected by shared underlying factors. Therefore, future research should adopt longitudinal designs to better clarify the temporal and causal relationships between mental health and Academic Tangping.

Lastly, Tangping is often perceived as negative, which necessitated careful handling of participant information to avoid bias and protect student privacy. Consequently, identifiable information was not collected, limiting the study’s ability to include longitudinal data. This oversight means that the research did not consider longitudinal measurement invariance or test-retest reliability, both of which are essential for validating the scale. Future research could address this by implementing strategies to protect student privacy, such as allowing participants to use pseudonyms or identification numbers. This approach would facilitate data collection at multiple time points, thereby enhancing the assessment of longitudinal measurement invariance and test-retest reliability.

##  Implications

This study has several important implications for teaching, student management, and future research. First, from a practical perspective, the Academic Tangping Scale can be used to identify high-risk student groups based on dimension-specific scores, enabling early and targeted intervention. Schools can classify students into high-, medium-, and low-risk groups according to their scores, prioritizing support for those most in need, while also using the data to monitor and evaluate intervention effectiveness over time. For instance, students exhibiting high affective tendencies may benefit from emotional regulation programs and individualized counseling, whereas those showing high-level behavioral tendencies can be supported through initiatives such as group-based projects, participation tracking, or mentor-guided academic monitoring. Similarly, students demonstrating cognitive tendencies may gain from study strategy training and goal-setting workshops aimed at enhancing self-directed learning and proactive academic planning. By linking interventions to the specific dimensions identified by the Academic Tangping Scale, educators can more effectively target support, identify high-risk student groups, and monitor the outcomes of such programs over time.

Second, from a theoretical perspective, the three-dimensional structure of the scale (affective, behavioral, cognitive) offers a novel approach for conceptualizing and measuring academic Tangping, advancing the theoretical understanding of the construct and providing a rigorous framework for future research on its dimensional structure and assessment.

Third, from a methodological perspective, the scale provides a valuable tool for future research. Researchers can explore the antecedents, developmental trajectories, and consequences of Academic Tangping, as well as its relationships with student well-being and academic performance, thereby advancing both theoretical understanding and evidence-based practice.

## Conclusion

In conclusion, this study has successfully developed and validated the Academic Tangping Scale, offering a comprehensive measure of the Tangping phenomenon across its affective, behavioral, and cognitive dimensions. The scale demonstrates strong reliability and validity, establishing it as a valuable tool for both educators and researchers. Ultimately, this research contributes to a deeper understanding of Tangping behavior and supports evidence-based strategies to foster student motivation and engagement in academic settings.

## Supplementary Information

Below is the link to the electronic supplementary material.

Supplementary Material 1

Supplementary Material 2

Supplementary Material 3

## Data Availability

The data supporting the findings of this study have been uploaded and are available for access. Please refer to the supplementary materials for further details.

## Appendix 2: Descriptive results

**Table:**

Variable	Mean		Std. Deviation Statistic	Pearson’s correlations																	
	Statistic	Std. Error		1. A1	2. A2	3. A3	4. A4	5. A5	6. B1	7. B2	8. B3	9. B4	10. B5	11. C1		12. C2	13. C3		14. C4	15. C5	
1. A1	3.280	0.058	1.050	—																	
2. A2	3.100	0.061	1.099	0.613	—																
3. A3	3.480	0.057	1.034	0.714	0.616	—															
4. A4	3.370	0.061	1.093	0.563	0.643	0.724	—														
5. A5	3.370	0.060	1.086	0.546	0.519	0.684	0.741	—													
6. B1	3.100	0.059	1.068	0.443	0.523	0.501	0.470	0.439	—												
7. B2	2.990	0.058	1.039	0.445	0.564	0.437	0.461	0.398	0.783	—											
8. B3	3.080	0.060	1.091	0.427	0.484	0.502	0.430	0.425	0.759	0.752	—										
9. B4	3.060	0.058	1.045	0.422	0.538	0.357	0.390	0.368	0.673	0.794	0.768	—									
10. B5	3.220	0.058	1.053	0.463	0.483	0.519	0.497	0.498	0.701	0.685	0.737	0.712	—								
11. C1	2.750	0.060	1.092	0.376	0.397	0.268	0.251	0.241	0.603	0.581	0.577	0.642	0.509	—							
12. C2	2.750	0.063	1.140	0.284	0.343	0.226	0.241	0.214	0.534	0.536	0.505	0.540	0.492	0.774		—					
13. C3	2.640	0.061	1.108	0.296	0.342	0.190	0.209	0.246	0.521	0.508	0.476	0.543	0.419	0.771		0.810	—				
14. C4	2.580	0.062	1.122	0.208	0.373	0.148	0.215	0.227	0.514	0.574	0.506	0.576	0.418	0.742		0.780	0.830		—		
15. C5	2.640	0.064	1.159	0.252	0.322	0.202	0.182	0.218	0.565	0.552	0.569	0.554	0.443	0.748		0.760	0.819		0.845	—	

Note: Correlations between variables are significant at the 0.01 level (2-tailed).
