# Supplementary material for: Academic Tangping scale for college students in China: scale development, validation and application
Source: Sci Rep. 2026 Feb 9;16:7897. doi: 10.1038/s41598-026-38759-2 (PMC12954069; doi:10.1038/s41598-026-38759-2)
Supplement: Supplementary file 3 — Supplementary Material 3 [file 41598_2026_38759_MOESM3_ESM.docx]

**Appendix 1**

**Academic Tangping Scale for College Students in China**

**Affective Factor**

**A1:** I am very satisfied with my current learning status, feeling at peace and not wanting to add too much pressure or anxiety to myself.

**A2:** I don’t have a strong desire or passion for achieving higher grades, and I feel content.

**A3:** I believe that maintaining inner peace and happiness is more important than striving for excellence, which makes me feel fulfilled.

**A4:** I am not interested in competition or surpassing others and prefer to study at my own pace, feeling comfortable and at ease.

**A5:** I care less about others' expectations and evaluations, focusing more on my own feelings and emotions to maintain my inner joy.

**Behavioral Factor**

**B1:** I attend classes on time and complete assignments, but I don’t actively pursue higher grades.

**B2:** I fulfill academic requirements but don’t invest extra time to improve my grades.

**B3:** I participate in necessary academic activities but don’t actively engage in competitions or additional training.

**B4:** I follow the teachers’ instructions in my studies, but I don’t actively seek knowledge beyond the course scope.

**B5:** I’d rather spend my free time on rest and personal interests than on extra studying.

**Cognitive Factor**

**C1:** I believe my abilities are limited, and effort won’t change the current situation.

**C2:** In my view, academic performance mainly depends on talent, and effort does not play a decisive role.

**C3:** I believe that fate is predetermined, and individual efforts cannot change the outcome.

**C4:** I feel that continuous effort only leads to more frustration, so it’s better to give up.

**C5:** I believe that no matter how hard I try, I won’t reach my ideal goals, so I choose not to push myself anymore.

**Note: All items are rated on a 5-point Likert scale (1 = Strongly Disagree, 5 = Strongly Agree).**
